# Comparison of in-brace correction between supine, molded, asymmetric TLSOs and standing, CAD, symmetric TLSOs

**DOI:** 10.1186/1748-7161-8-S1-O40

**Published:** 2013-06-03

**Authors:** D Speers

**Affiliations:** 1 Scheck and Siress Orthotics and Prosthetics, Chicago, IL, USA

## Background

Bracing for adolescent idiopathic scoliosis is a major component of conservative care. Many different brace designs exist, but they all must place corrective forces on the apex of the curves.

## Aim

To determine whether asymmetric braces, molded supine, with manipulation during casting, compared to symmetric CAD designs, measured standing, offered better in brace correction.

## Methods

34 females (ages 7 – 14) with adolescent idiopathic scoliosis were treated with a TLSO, and initial in brace radiographs were evaluated for correction of the curves. 22 received a symmetrical, posterior open CAD design made from standing measurements, and 12 received an asymmetric, posterior open design made from a supine casting with correction of the curve(s) applied during casting.

## Results

For the 22 symmetric CAD TLSOs, initial in brace correction of thoracic curves was 9.1 degrees, with average out of brace thoracic curve 25.2 degrees, and lumbar curve correction of 10.3 degrees, with average out of brace lumbar curve 27.8 degrees. The percentage of correction in CAD versions was 36.1% thoracic, and 37.1% lumbar.

For the 12 asymmetric, TLSOs molded supine with manipulation, initial in brace correction of thoracic curves was 13.3 degrees, with average out of brace thoracic curve 26.7 degrees, and lumbar curve correction of 12.1 degrees, with average out of brace lumbar curve 26.6 degrees. The percentage of correction in supine, molded TLSOs was 49.8% thoracic, and 45.5% lumbar.

## Conclusion

Supine, molded posterior open TLSOs with correction during casting provide greater initial in brace correction of both lumbar and thoracic curves compared to posterior open, symmetric, CAD designs made from standing measurements.
